# Visfatin Induces Sickness Responses in the Brain

**DOI:** 10.1371/journal.pone.0015981

**Published:** 2011-01-20

**Authors:** Byong Seo Park, Sung Ho Jin, Joong Jean Park, Jeong Woo Park, Il Seong Namgoong, Young Il Kim, Byung Ju Lee, Jae Geun Kim

**Affiliations:** 1 Department of Biological Sciences, College of Natural Sciences, University of Ulsan, Ulsan, Republic of Korea; 2 Biomedical Research Center, College of Medicine, University of Ulsan, Ulsan, Republic of Korea; 3 Department of Physiology, College of Medicine, Korea University, Seoul, Republic of Korea; 4 Department of Internal Medicine, Ulsan University Hospital, Ulsan, Republic of Korea; University of Camerino, Italy

## Abstract

**Background/Objective:**

Visfatin, also known as nicotiamide phosphoribosyltransferase or pre-B cell colony enhancing factor, is a pro-inflammatory cytokine whose serum level is increased in sepsis and cancer as well as in obesity. Here we report a pro-inflammatory role of visfatin in the brain, to mediate sickness responses including anorexia, hyperthermia and hypoactivity.

**Methodology:**

Rats were intracerebroventricularly (ICV) injected with visfatin, and changes in food intake, body weight, body temperature and locomotor activity were monitored. Real-time PCR was applied to determine the expressions of pro-inflammatory cytokines, proopiomelanocortin (POMC) and prostaglandin-synthesizing enzymes in their brain. To determine the roles of cyclooxygenase (COX) and melanocortin in the visfatin action, rats were ICV-injected with visfatin with or without SHU9119, a melanocortin receptor antagonist, or indomethacin, a COX inhibitor, and their sickness behaviors were evaluated.

**Principal Findings:**

Administration of visfatin decreased food intake, body weight and locomotor activity and increased body temperature. Visfatin evoked significant increases in the levels of pro-inflammatory cytokines, prostaglandin-synthesizing enzymes and POMC, an anorexigenic neuropeptide. Indomethacin attenuated the effects of visfatin on hyperthermia and hypoactivity, but not anorexia. Further, SHU9119 blocked visfatin-induced anorexia but did not affect hyperthermia or hypoactivity.

**Conclusions:**

Visfatin induced sickness responses via regulation of COX and the melanocortin pathway in the brain.

## Introduction

Sickness responses such as anorexia and changes in energy metabolism are closely related to inflammatory diseases [1], [2]. Inflammation-associated anorexia refers to reduced food intake during acute and chronic inflammatory states in both human and animals. It is well known that laboratory animals reduce their food intake in response to administration of pro-inflammatory cytokines or agents that stimulate cytokine release such as lipopolysaccharide (LPS) [3], [4], [5].

Inflammation affects the central nervous system and results in the manifestation of sickness symptoms [6]. Inflammation in the brain elicits a state of profound negative energy balance that is an adaptive response to infection [1] and induces sickness responses such as fever, anorexia, weakness and hypoactivity [2]. A number of inflammatory stimuli activate hypothalamic pro-inflammatory cytokines, including tumor necrosis factor-α (TNF- α), interleukin 1-β (IL1-β) and IL-6, which is involved in anorexia and febrile responses. Conversely, inhibition of cytokine production or action attenuates these inflammation-induced sickness responses [7], [8]. Recent studies have suggested that synthesis and release of pro-inflammatory cytokines in response to pathophysiological processes induce anorexia and increase metabolic rate by acting upon the brain region responsible for energy homeostasis [9], [10].

Visfatin has been recently identified as a peptide predominantly expressed in and secreted from visceral fat in both humans and mice [11], [12]. This protein is also known as an enzyme for the biosynthesis of NAD^+^, which influences a variety of metabolic and stress responses [13]. Although recent studies have emphasized its role as an adipose hormone that mediates pro-inflammatory actions in various metabolic diseases like obesity, type 2 diabetes and cardiovascular disease [13], visfatin was originally identified as a pre-B cell colony enhancing factor (PBEF) and is thought to play roles in immune response and inflammation [14], [15], [16], [17]. Thus, there is some evidence to suggest that visfatin activates pro-inflammatory cytokines in human monocytes [18]. Additionally, serum visfatin concentration is increased in patients with sepsis, chronic kidney disease and cancer [19], [20], [21], which indicates that visfatin plays a pro-inflammatory role in peripheral tissues. However, little is known about its function in the brain.

Accordingly, the aim of the present study is to identify the roles of visfatin in energy metabolism and in sickness responses in the brain. We assessed changes in food intake, body temperature and locomotor activity after intracerebroventricular (ICV) administration of visfatin and identified the molecular mechanisms of these physiological responses.

## Results

### Effects of visfatin on food intake and body weight

**Figure 1:**
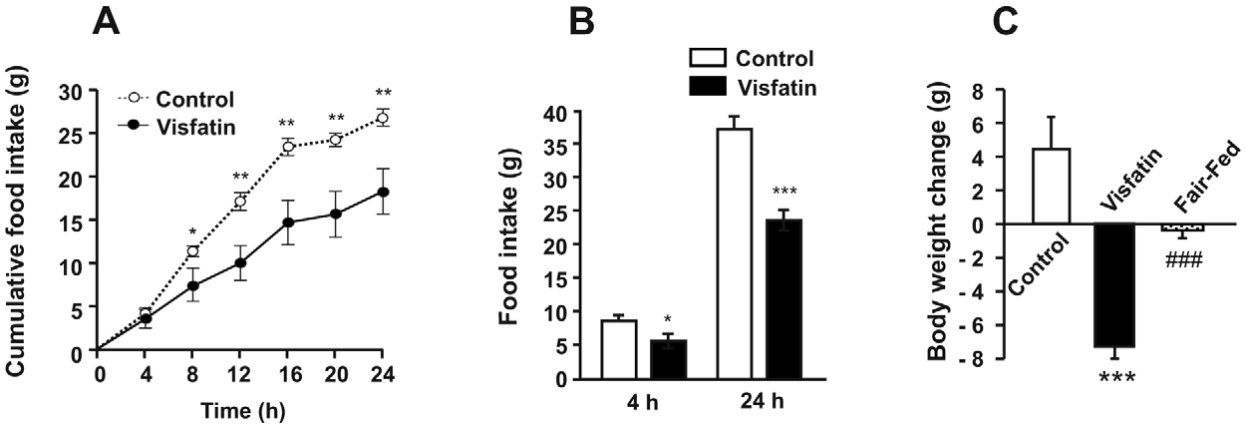
Effects of ICV administration of visfatin on food intake and body weight. Food intake and body weight were measured in rats that had received an ICV injection of visfatin. (A) Cumulative food intake was measured for one day at 4 h intervals after an ICV injection of visfatin. Control animals received a 0.9% saline solution. (B) Effect of visfatin on food intake in rats showing strong appetites induced by food deprivation for one day. (C) Effect of visfatin on body weight changes one day after ICV injection. Data are represented as mean ± SEM (n = 10). *P<0.05, **P<0.01 and ***P<0.001 vs. control rats; ^###^P<0.001 vs. visfatin-injected rats.

In order to assess the central role of visfatin in energy homeostasis, rats were injected with recombinant rat visfatin into the lateral ventricle. Administration of visfatin significantly decreased cumulative food intake measured at 4-h intervals for 24 h after injection of visfatin (Fig. 1A). Significant differences were observed between the visfatin- and vehicle-injected groups beginning 8 h after visfatin injection. To confirm the effect of visfatin on feeding behavior, visfatin was ICV-injected into animals that had fasted for 24 h, in whom a strong appetite was induced. Visfatin significantly decreased food intake 4 and 24 h after re-feeding in food-deprived animals (Fig. 1B). To determine if the anorectic effect of visfatin affects body weight, rats was weighed immediately prior to visfatin injection and 24 h after the injection. Control animals showed an average of 4.4 g of weight gain during the first day, while visfatin-injected rats lost an average of 7.3 g (Fig. 1C). Interestingly, weight loss in visfatin-injected rats was significantly greater than that of the pair-fed group that was given the same amount of food. This suggests that central administration of visfatin may have affected energy expenditure as well as food intake.

### Visfatin-induced hyperthermia and hypoactivity

**Figure 2:**
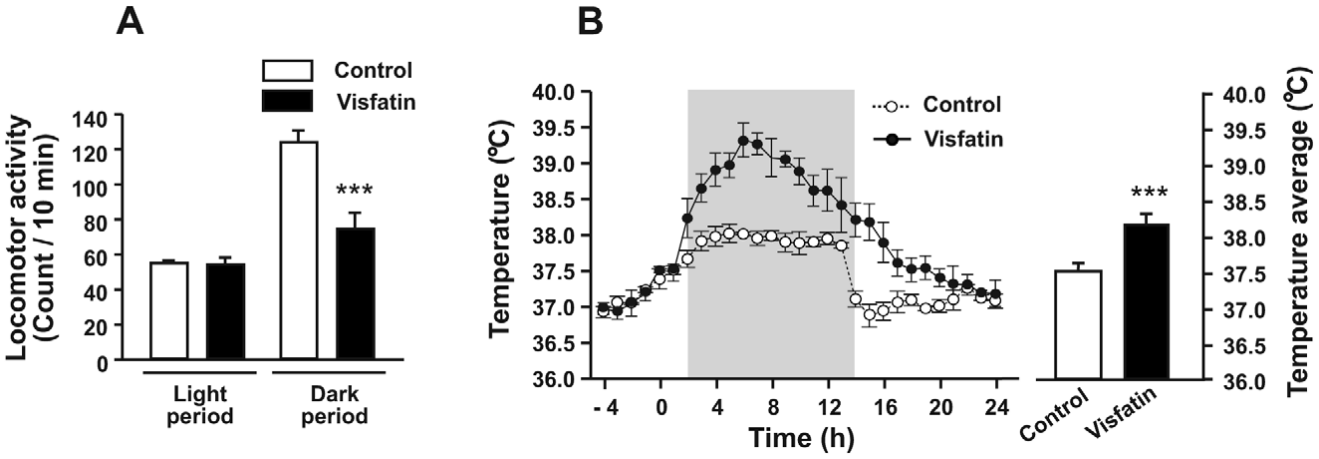
Effects of visfatin on locomotor activity and body temperature. Locomotor activity and body temperature were measured for 24 h after ICV administration of visfatin. (A) ICV injection of visfatin resulted in decreased locomotor activity during the dark period, but there was no change in activity during the light period. (B) Body temperature began to increase 2 h after ICV injection of visfatin and remained high until about 20 h after injection. Mean temperature after the injection time (at 0 h) was significantly different between groups. Data are represented as mean ± SEM (n = 10). ***P<0.001 vs. control rats injected with 0.9% saline solution.

We evaluated body temperature and locomotor activity in rats injected with visfatin. As shown in Fig. 2A, locomotor activity in visfatin-treated rats during the dark period was significantly lower than that of vehicle-treated rats. Vehicle-treated control rats showed circadian changes in body temperature: low during the light period and high during the dark period. Rats administered visfatin 2 h before the dark period showed an increase in body temperature beginning 2 h after injection and peaking 8 h after injection (Fig. 2B). This increase in body temperature continued until the start of the light period.

### Visfatin stimulates hypothalamic factors involved in inflammatory responses

**Figure 3:**
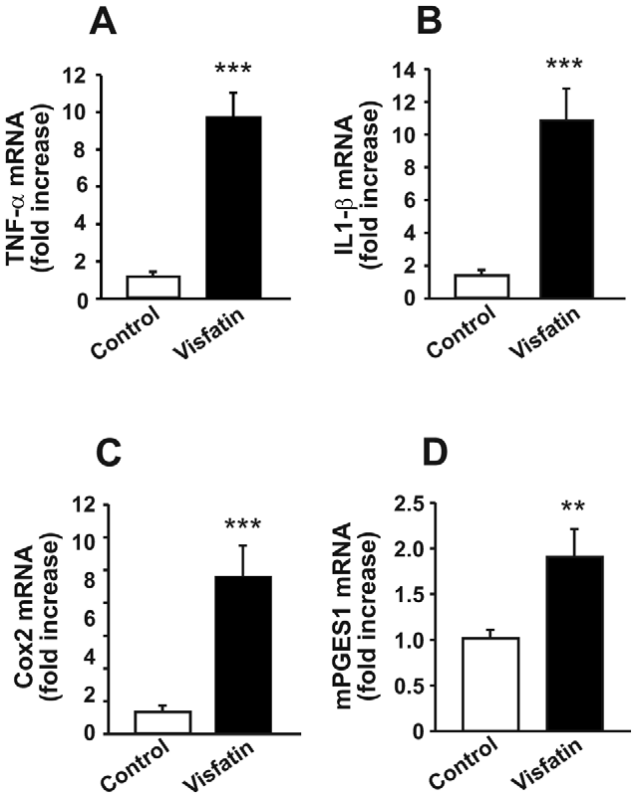
Visfatin-induced increases in hypothalamic mRNA levels of pro-inflammatory cytokines and prostaglandin-synthesizing enzymes. RNA was extracted from rat hypothalami 6 h after ICV injection of visfatin. mRNA expressions encoding TNF-α, IL-1β, COX2 and mPGES-1 were determined using real-time PCR. Visfatin significantly stimulated the expressions of TNF-α (A), IL-1β (B), COX2 (C) and mPGES-1 (D) mRNA in the hypothalamus. Data are represented as mean ± SEM (n = 6). **P<0.01 and ***P<0.001 vs. control rats injected with 0.9% saline solution.

To determine whether visfatin is involved in hypothalamic inflammation, we assessed its effect on the expressions of pro-inflammatory cytokines and prostaglandin-synthesizing enzymes in the rat hypothalamus. ICV administration of visfatin increased the expressions of TNF-α and IL1-β in the hypothalamus (Figs. 3A and B). Moreover, there were significant increases in cyclooxygenase 2 (COX2), a rate-limiting enzyme that converts arachidonic acid into prostaglandins, and microsomal prostaglandin E synthase-1 (mPGES1), a specific catalyzing enzyme for the final step of prostaglandin E2 (PGE2) biosysthesis (Figs. 3C and D). These results suggest that visfatin may be involved in an inflammatory response in the brain by activating the syntheses of inflammatory mediators.

### COX inhibitor blocks visfatin-induced hyperthermia and hypoactivity, but not anorexia

**Figure 4:**
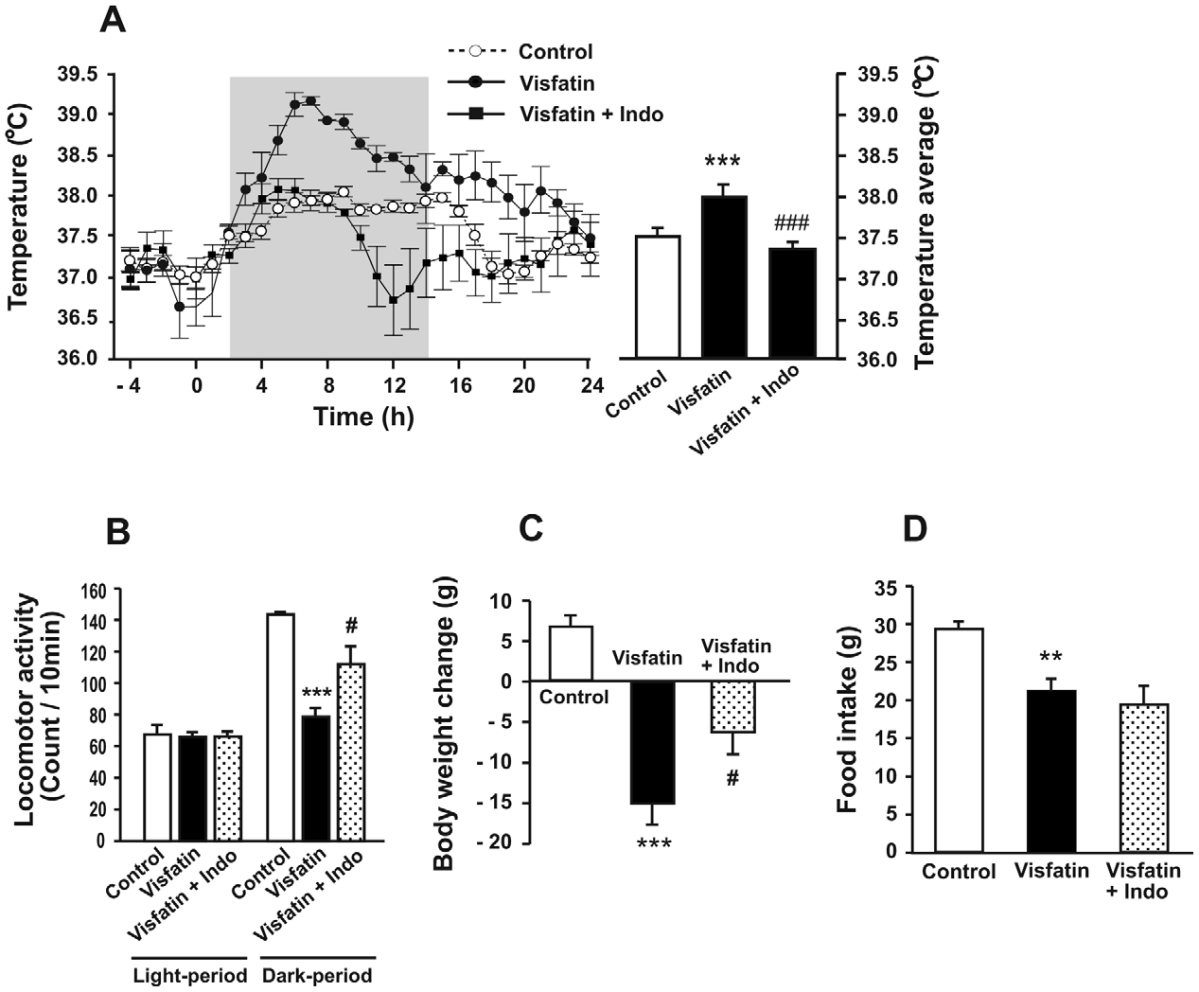
Effect of COX inhibitor on visfatin-induced sickness behaviors. To determine the involvement of prostaglandins on visfatin-induced sickness behaviors, rats were IP-injected with indomethacin (Indo) 30 min prior to injection with visfatin. Parameters such as body temperature, locomotor activity and food intake were observed for one day after injection of visfatin. Indomethacin completely blocked the visfatin-induced increase in body temperature (A) and partially attenuated the effects of visfatin on locomotor activity (B) and body weight (C). However, indomethacin did not affect the visfatin-induced decrease in food intake (D). Data are represented as mean ± SEM (n = 6). **P<0.01 and ***P<0.001 vs. control rats injected with 0.9% saline solution; ^#^P<0.05 and ^###^P<0.001 vs. visfatin-injected rats.

Because we found that visfatin stimulated the expressions of COX2 and mPGES1 genes in the hypothalamus, we hypothesized that visfatin might exert its effect on sickness behaviors by regulating the syntheses of prostaglandins. To investigate this possibility, rats were intraperitoneally (IP) injected with indomethacin, a COX inhibitor, 30 min prior to the injection of visfatin, and their sickness responses were observed. Indomethacin completely abolished visfatin-induced hyperthermia during the observation period (Fig. 4A), and visfatin-induced hypoactivity (Fig. 4B) and weight loss (Fig. 4C) were partially blocked by the same treatment. However, indomethacin did not affect visfatin-induced decrease of food intake (Fig. 4D). These results suggest that visfatin affects body temperature and locomotor activity via prostaglandin activities. Additionally, its effect on food intake may be mediated by a different pathway.

### Effect of visfatin on α-melanocyte stimulating hormone (α –MSH) synthesis

**Figure 5:**
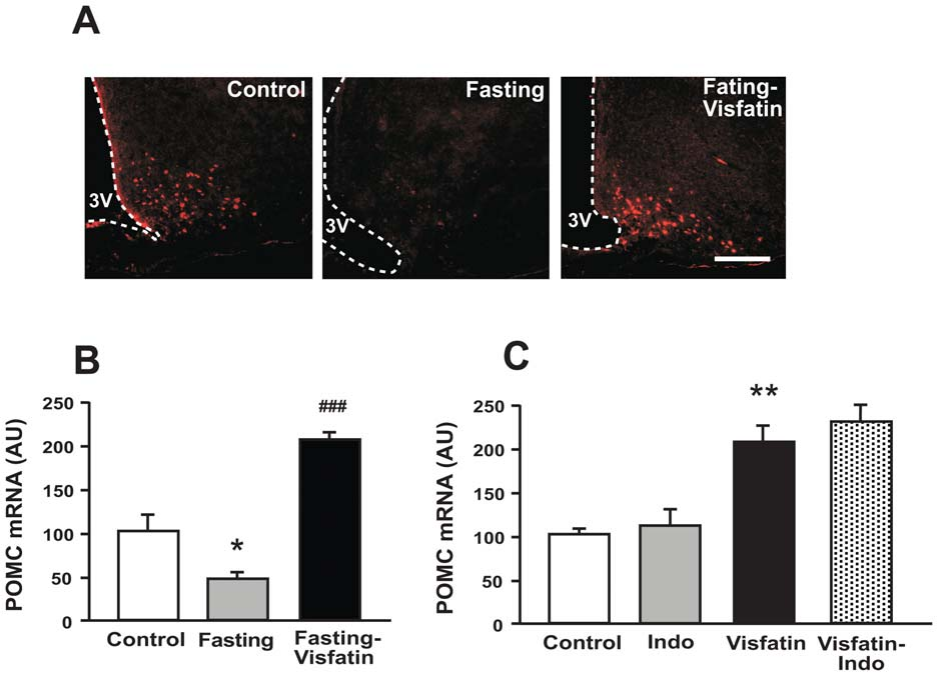
Effect of ICV administration of visfatin on α-MSH synthesis in the ARC. To determine the effect of visfatin on α-MSH synthesis, visfatin was ICV-injected into the lateral ventricles of rats deprived of food for one day. Ninety minutes after the injection, the rats were sacrificed and their brains were fixed via transcardiac perfusion for IHC or were sliced for excising the ARC using a micropunch. Expressions of α-MSH (A) and POMC (B) were determined through IHC and real-time PCR, respectively. To determine the effects of prostaglandins on visfatin-induced changes in POMC expression, indomethacin (Indo) was IP-injected 30 min prior to injection of visfatin, and its effect was determined using real-time PCR (C). 3V = third ventricle. Data (in B and C) are represented as mean ± SEM (n = 6). *P<0.05 and **P<0.01 vs. control rats injected with 0.9% saline solution; ^###^P<0.001 vs. fasting. Scale bar  = 50 µm.

To investigate whether visfatin exerts an anorexic effect by regulating the hypothalamic melanocortin pathway that is well-known for controlling appetite, hypothalamic expressions of α-MSH and proopiomelanocortin (POMC) were determined in rats injected with visfatin using immunohistochemistry (IHC) and real-time PCR, respectively. Food deprivation for one day significantly decreased α-MSH protein (Fig. 5A) and POMC mRNA (Fig. 5B) levels in the hypothalamic arcuate nucleus (ARC), in agreement with prior findings [22]. Interestingly, visfatin completely reversed the effect of fasting and the subsequent decreases in α-MSH protein and POMC mRNA levels. In fact, visfatin increased the expressions of both peptides compared to those of untreated control rats (Figs. 5A and B). However, indomethacin did not affect vsifatin-induced enhanced POMC expression (Fig. 5C). These results suggest that the anorexic effects of visfatin may be caused by its effect on the syntheses of POMC and α-MSH and not via prostaglandins.

### Effect of a melanocortin receptor antagonist on visfatin-induced anorexia

**Figure 6:**
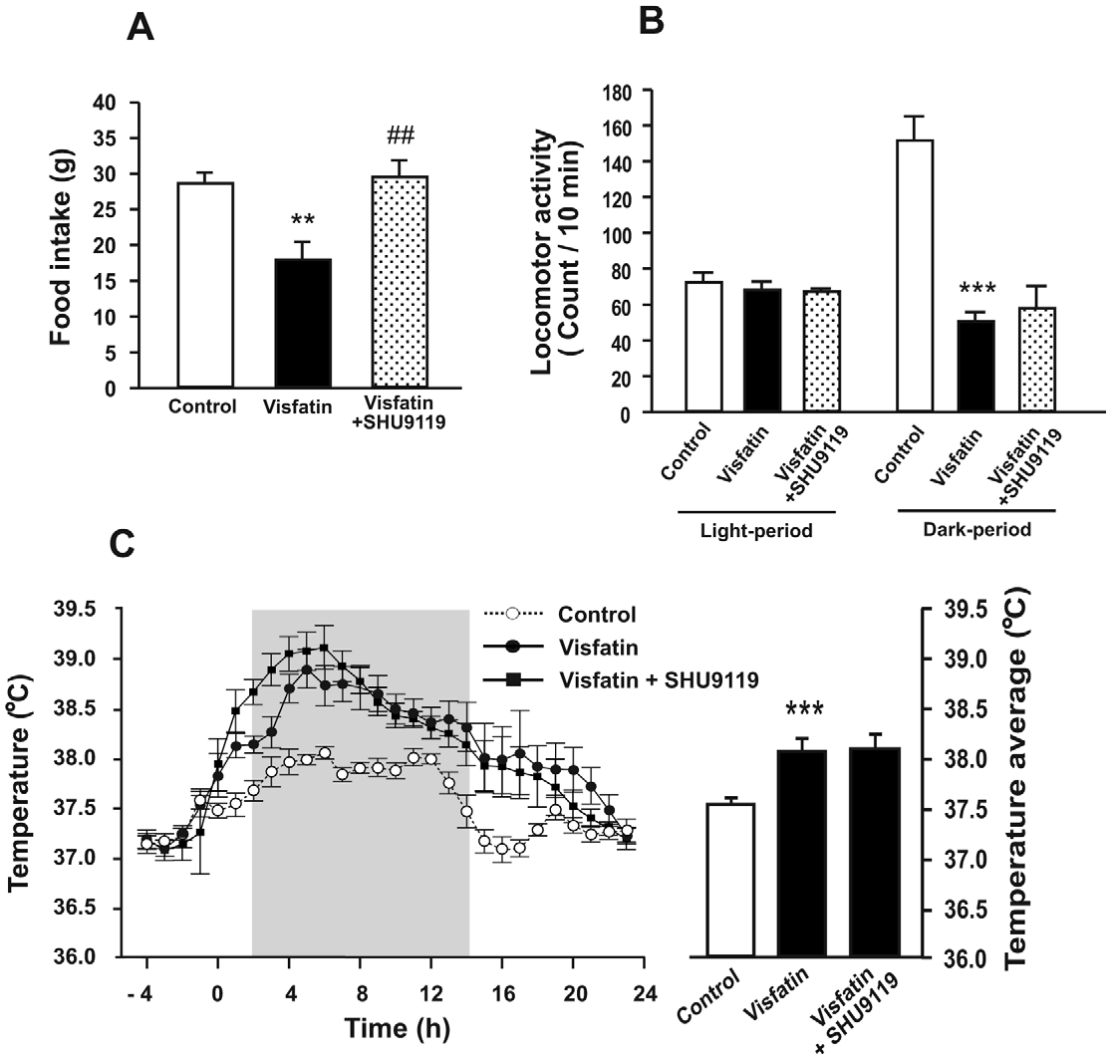
Effects of melanocortin receptors 3/4 antagonist on visfatin-induced sickness behaviors. Two-month-old male rats were ICV-injected with visfatin or vehicle 30 min after ICV injection of SHU9119 and were allowed access to food immediately after the final treatment. (A) Change in food intake was measured 24 h after the injections of SHU9119 and visfatin. (B, C) Changes in locomotor activity (B) and body temperature (C) were monitored in rats IP-implanted with telemetry-transmitters. In C, time 0 indicates visfatin injection. Data are represented as mean ± SEM (n = 6). **P<0.01 and ***P<0.001 vs. control rats injected with 0.9% saline solution; ^##^P<0.01 vs. visfatin-injected rats.

It has been well established that α-MSH decreases food intake by acting through melanocortin receptors 3 and 4 (MC3/4R) [23]. Accordingly, we examined whether the anorectic effect of visfatin is mediated by MC3/4R using rats ICV-injected with SHU9119, an MC3/4R antagonist. When SHU9119 was pre-administered to rats 30 min prior to the visfatin injection, it completely blocked the anorectic effect of visfatin (Fig. 6A), indicating that visfatin-induced anorexia is mediated by MC3/4R. Next, we examined the effects of SHU9119 on visfatin-induced hypoactivity and hyperthermia in the same group of rats. ICV administration of SHU9119 did not change the effect of visfatin on locomotor activity (Fig. 6B) or body temperature during the 24-h observation period (Fig. 6C). However, it further increased body temperature from the visfatin-increased level during the initial 8 h following injection of SHU9119 (Fig. 6C). The areas under the curves of the two groups (visfatin and visfatin + SHU9119) significantly differed during this period (P<0.001).

## Discussion

Since the discovery of visfatin expression in visceral fat, several studies have reported correlations between serum visfatin concentration and expression with obesity, but the relationship between obesity and plasma visfatin has not been well established. Some studies have reported higher plasma visfatin levels in obese individuals [11], [24], whereas other studies have reported opposing findings [12], [25].

The present study evaluated the effects of visfatin on food intake and body weight to clarify the relationship between visfatin and obesity. We found that ICV-injected visfatin decreased food intake and body weight. Moreover, we observed that this peptide induced a much greater decrease in body weight compared to that of a matched, pair-fed group, suggesting that one of the main roles of visfatin is in the processes of energy consumption. Surprisingly, ICV-visfatin injections decreased the locomotor activities of rats. We also found that visfatin dramatically increased body temperature. Collectively, these results suggest that visfatin is involved in a sickness response in the brain, as increased body temperature and decreased locomotor activity are representative indicators of an inflammatory sickness response.

We also found that pro-inflammatory cytokines (TNF-α and IL1-β) and PGE2-synthesizing enzymes (COX2 and mPGES1) were profoundly stimulated by visfatin. Recent studies have suggested that visfatin may have a role in the regulation of peripheral inflammatory responses [16], [17]. Serum visfatin concentration is also increased in patients with inflammatory diseases like chronic kidney disease, sepsis and cancer [19], [20], [21]. Additionally, visfatin activates other pro-inflammatory cytokines as well, including IL-6, IL-1β and TNF-α in human monocytes [18]. A key mediator for inflammatory process, PGE2 plays an important role in the development of sickness behaviors observed during inflammatory states [5]. In the brain, PGE2 is produced by a variety of inflammatory signals such as endotoxins or cytokines and is one of the critical inducers of sickness responses [26]. Previous research has also found that COX2 is involved in an inflammatory response. Selective pharmacological or genetic blockade of COX2 effectively attenuates the sickness response to systemic inflammation induced by LPS, a cell wall component of gram-negative bacteria [5], [27]. Collectively, these results strongly suggest that visfatin may be involved in inflammatory sickness responses by regulating the productions of hypothalamic pro-inflammatory cytokines and prostaglandins.

To identify pathways of visfatin action in the control of sickness responses, we examined the effect of indomethacin, a COX inhibitor, on sickness behaviors in response to visfatin. Indomethacin completely abolished visfatin-induced hyperthermia and partially reversed visfatin-induced hypoactivity but did not affect visfatin-induced anorexia. This result is unexpected because COX inhibitors are known to reverse decreased food intake induced by inflammatory conditions [27]. In this study, we found that visfatin increased the synthesis of α-MSH, an anorectic neuropeptide in normal and fast-induced hyperphagic conditions. It may be that visfatin-induced anorexia is due in part to effects of the central melanocortin pathway, which plays an important role in the mediation of anorexia and cachexia [10]. Indeed, we found that blocking melanocortin receptors with SHU9119, a MC3/4R antagonist, attenuated the anorectic effect of visfatin. Thus, the melanocortin pathway is a likely mediator of visfatin effects on feeding behavior. Additionally, we found that administration of SHU9119 did not block visfatin-induced hyperthermia or hypoactivity, but that it rather exerted an immediate additional increase in body temperature. Interestingly, this immediate increased body temperature by SHU9119 well coincides with a previous study showing that central administration of SHU9119 exacerbated LPS-induced fever during the period 0–8 h after injection of LPS, but not during subsequent intervals, while it did not affect LPS-induced hypoactivity [28]. Thus, evidence suggests that the hypothalamic melanocortin pathway is not a critical mediator for visfatin-induced hyperthermia and hypoactivity. Our results are also in line with those of previous studies that have found that ICV injection of low doses of α-MSH inhibits hyperthermia that is induced by endotoxins or cytokines [28]. Collectively, such results suggest that visfatin acts on food intake via the melanocortin pathway, and it acts on hyperthermia and hypoactivity mainly via COX.

In summary, our results indicate that visfatin is a regulator of behavioral responses to sickness and acts as a classic inflammatory signal to activate responses to acute inflammation. To our knowledge, our study is one of the first to differentiate two pathways of action for visfatin-induced anorexia, hypothermia, and hypoactivity.

## Materials and Methods

### Animals

Two-month-old male Sprague-Dawley rats (Daehan Animal Breeding Company, Chungwon, Korea) were used, and animal experiments were conducted in accordance with the regulations of the University of Ulsan and the National Institutes of Health Guide for the Care and Use of Laboratory Animals. The Institutional Review Board of University of Ulsan approved the experimental procedures (permission number UOU-2008-07). Rats were housed in a room with a conditioned photoperiod (12 h light/12 h dark, lights on from 0600-1800) and a regulated temperature (23–25°C) and were allowed *ad libitum* access to tap water and rat chow pellets.

### Micropunch dissection

Rats were sacrificed via decapitation, and their brains were rapidly removed and frozen in 2-methylbutane on dry ice for 5 min. Brains were sectioned (500-µm thickness) in a cryostat at -15°C and were mounted onto glass slides. Using anatomical landmarks from the rat brain atlas, the ARC was identified and punched out under a stereomicroscope using a micro-punching set (Stoelting, Wood Dale, IL). Micro-punched ARC fragments were stored in microcentrifuge tubes at -80°C.

### Real-time PCR

Total RNA was isolated from the total hypothalamus and micro-punched ARC using Trizol reagent (Sigma-Aldrich). The samples were reverse-transcribed and amplified using real-time PCR with the following primer sets: POMC sense primer, 5′-GCT AGG TAA CAA ACG AAT GG-3′; antisense primer, 5′-GCA TTT TCT GTG CTT TCT CT-3′; TNF-α sense primer, 5′- AAA GCA TGA TCC GAG ATG TG-3′; antisense primer, 5′- AGC AGG AAT GAG AAG AGG CT-3′; IL-1β sense primer, 5′- CAT CTT TGA AGA AGA GCC CG-3′; antisense primer, 5′- GGG ATT TTG TCG TTG CTT GT-3′; COX2 sense primer, 5′-ACC AGA GCA GAG AGA TGA AA-3′; antisense primer, 5′-GAG AGA CTG AAT TGA GGC AG-3′; mPGES1 sense primer, 5′-CTG CTG GTC ATC AAG ATG TAC G-3′; antisense primer, 5′-CCC AGG TAG GCC ACG GTG TGT-3′; glyceraldehyde-3-phosphate dehydrogenase (GAPDH) sense primer, 5′-TGT GAA CGG ATT TGG CCG TA-3′; and antisense primer, 5′-ACT TGC CGT GGG TAG AGT CA-3′. Real-time PCR was carried out in capillaries of the DNA Engine Opticon Continuous Fluorescence Detection System (MJ Research Inc., Waltham, MA) for approximately 40 cycles as follows: at 94°C for 30 sec, 56°C for 30 sec and 72°C for 35 sec.

### Immunohistochemistry (IHC)

Rats were anesthetized with tribromoethanol (250 mg/kg B.W., Sigma-Aldrich) and transcardially perfused with 100 ml ice-cold 0.1M phosphate buffer (PB, pH 7.4), followed by 100 ml 4% paraformaldehyde. Brains were dissected and post-fixed overnight in the same fixative containing 30% sucrose. Slide-mounted sections were prepared with a cryostat. We followed the IHC protocol described previously [29], using a primary antibody for α-MSH (1∶10000; Millipore, Billerica, MA), and secondary antibody for sheep IgG (1∶500; Vector, Burlingame, CA). Immunoreactive signals were visualized using the Tyramide Signal Amplification System (NEN Life Science, Boston, MA), and images were obtained using fluorescence microscopy.

### Stereotaxic surgery for intracerebroventricular cannulae

Rats were anesthetized via an IP injection of tribromoethanol (250 mg/kg B.W., Sigma-Aldrich) and were placed in a stereotaxic apparatus (Stoelting, Wood Dale, IL). A polyethylene cannula (o.d. 1.05 mm, i.d. 0.35 mm) was implanted into the lateral ventricle (coordinates: AP = 1.0 mm caudal to the bregma; V = 3.6 mm from the dura mater; L = 0.16 mm from the mid-line) and secured to the skull with dental cement. The rats were immediately sutured and placed in individual cages. After one week of recovery, test materials were injected through the cannula.

### ICV administration of visfatin

To determine whether visfatin regulates sickness responses in the brain, rats were ICV-injected with recombinant rat visfatin (2 µg, Adipogene, Seoul, Korea). Rats were sacrificed 90 min or 6 h after visfatin injection. The hypothalamus was dissected and the RNA was isolated.

### Treatment of COX inhibitor and MC3/4R antagonist

To determine whether MC3/4R and COX pathways mediate sickness behaviors induced by the ICV administration of visfatin, we pre-injected SHU9119 (an MC3/4R antagonist; Phoenix Pharmaceuticals, Burlingame, CA) and indomethacin (a COX inhibitor; Sigma-Aldrich) 30 min prior to visfatin injection. Immediately after injection, rats were allowed *ad libitum* access to food, and their cumulative food intake was measured 24 h after the injection. To test the effects of SHU9119 and indomethacin on visfatin-induced hyperthermia and hypoactivity, body temperature and locomotor activity were measured using telemetry transmitters implanted into the rats following the procedures described above.

### Measurement of body temperature and locomotor activity

Abdominal temperature and locomotor activity were measured in male Sprague-Dawley rats using biotelemetry transmitters (Mini-Mitter, Bend, OR) implanted into the abdominal cavity one week prior to the experiment. Prior to surgery, rats were anesthetized with tribromoethanol (250 mg/kg B.W., Sigma-Aldrich). The output (frequency in Hz) was monitored by a receiver (model RA 1000; Mini-Mitter) placed under each cage. A data acquisition system (Vital View; Mini-Mitter) was used for automatic control of data collection and analysis. Body temperature was recorded at 10-min intervals. Changes in locomotor activity were detected as changes in the position of the implanted transmitter over the receiver board, which resulted in a change in the signal strength and was recorded as a pulse of activity. Activity pulses were counted every 10 min and were summed after 12 h as a cumulative measure of daytime or nighttime activity. Locomotor activity scores are expressed as activity counts per 12 h.

### Statistics

All results are expressed as mean ± standard error of measure (SEM; n is given in the figure legends). For cumulative food intake, statistical analyses were performed using repeated measures ANOVA with Bonferroni post-hoc analyses. Student's t-test was used for comparison of two groups.

## References

[1] Kluger MJ (1991). Fever: role of pyrogens and cryogens.. Physiol Rev.

[2] Skinner GW, Mitchell D, Harden LM (2009). Avoidance of physical activity is a sensitive indicator of illness.. Physiol Behav.

[3] Reyes TM, Sawchenko PE (2002). Involvement of the arcuate nucleus of the hypothalamus in interleukin-1-induced anorexia.. J Neurosci.

[4] Harden LM, du Plessis I, Poole S, Laburn HP (2008). Interleukin (IL)-6 and IL-1 beta act synergistically within the brain to induce sickness behavior and fever in rats.. Brain Behav Immun.

[5] Pecchi E, Dallaporta M, Jean A, Thirion S, Troadec JD (2009). Prostaglandins and sickness behavior: old story, new insights.. Physiol Behav.

[6] Elmquist JK, Scammell TE, Saper CB (1997). Mechanisms of CNS response to systemic immune challenge: the febrile response.. Trends Neurosci.

[7] Konsman JP, Parnet P, Dantzer R (2002). Cytokine-induced sickness behaviour: mechanisms and implications.. Trends Neurosci.

[8] Thaler JP, Choi SJ, Schwartz MW, Wisse BE (2010). Hypothalamic inflammation and energy homeostasis: resolving the paradox.. Front Neuroendocrinol.

[9] Andreasson A, Arborelius L, Erlanson-Albertsson C, Lekander M (2007). A putative role for cytokines in the impaired appetite in depression.. Brain Behav Immun.

[10] Laviano A, Inui A, Marks DL, Meguid MM, Pichard C (2008). Neural control of the anorexia-cachexia syndrome.. Am J Physiol Endocrinol Metab.

[11] Berndt J, Kloting N, Kralisch S, Kovacs P, Fasshauer M (2005). Plasma visfatin concentrations and fat depot-specific mRNA expression in humans.. Diabetes.

[12] Mercader J, Granados N, Caimari A, Oliver P, Bonet ML (2008). Retinol-binding protein 4 and nicotinamide phosphoribosyltransferase/visfatin in rat obesity models.. Horm Metab Res.

[13] Imai S (2009). Nicotinamide phosphoribosyltransferase (Nampt): a link between NAD biology, metabolism, and diseases.. Curr Pharm Des.

[14] Samal B, Sun Y, Stearns G, Xie C, Suggs S (1994). Cloning and characterization of the cDNA encoding a novel human pre-B-cell colony-enhancing factor.. Mol Cell Biol.

[15] Luk T, Malam Z, Marshall JC (2008). Pre-B cell colony-enhancing factor (PBEF)/visfatin: a novel mediator of innate immunity.. J Leukoc Biol.

[16] Oki K, Yamane K, Kamei N, Nojima H, Kohno N (2007). Circulating visfatin level is correlated with inflammation, but not with insulin resistance.. Clin Endocrinol (Oxf).

[17] Moschen AR, Gerner RR, Tilg H (2010). Pre-B cell colony enhancing factor/NAMPT/visfatin in inflammation and obesity-related disorders.. Curr Pharm Des.

[18] Moschen AR, Kaser A, Enrich B, Mosheimer B, Theurl M (2007). Visfatin, an adipocytokine with proinflammatory and immunomodulating properties.. J Immunol.

[19] Jia SH, Li Y, Parodo J, Kapus A, Fan L (2004). Pre-B cell colony-enhancing factor inhibits neutrophil apoptosis in experimental inflammation and clinical sepsis.. J Clin Invest.

[20] Carrero JJ, Witasp A, Stenvinkel P, Qureshi AR, Heimburger O (2010). Visfatin is increased in chronic kidney disease patients with poor appetite and correlates negatively with fasting serum amino acids and triglyceride levels.. Nephrol Dial Transplant.

[21] Nakajima TE, Yamada Y, Hamano T, Furuta K, Gotoda T (2009). Adipocytokine levels in gastric cancer patients: resistin and visfatin as biomarkers of gastric cancer.. J Gastroenterol.

[22] Sainsbury A, Zhang L (2010). Role of the arcuate nucleus of the hypothalamus in regulation of body weight during energy deficit.. Mol Cell Endocrinol.

[23] Shimizu H, Inoue K, Mori M (2007). The leptin-dependent and -independent melanocortin signaling system: regulation of feeding and energy expenditure.. J Endocrinol.

[24] Sun G, Bishop J, Khalili S, Vasdev S, Gill V (2007). Serum visfatin concentrations are positively correlated with serum triacylglycerols and down-regulated by overfeeding in healthy young men.. Am J Clin Nutr.

[25] Pagano C, Pilon C, Olivieri M, Mason P, Fabris R (2006). Reduced plasma visfatin/pre-B cell colony-enhancing factor in obesity is not related to insulin resistance in humans.. J Clin Endocrinol Metab.

[26] Rorato R, Menezes AM, Giusti-Paiva A, de Castro M, Antunes-Rodrigues J (2009). Prostaglandin mediates endotoxaemia-induced hypophagia by activation of pro-opiomelanocortin and corticotrophin-releasing factor neurons in rats.. Exp Physiol.

[27] Johnson PM, Vogt SK, Burney MW, Muglia LJ (2002). COX-2 inhibition attenuates anorexia during systemic inflammation without impairing cytokine production.. Am J Physiol Endocrinol Metab.

[28] Huang QH, Hruby VJ, Tatro JB (1999). Role of central melanocortins in endotoxin-induced anorexia.. Am J Physiol.

[29] Kim JG, Nam-Goong IS, Yun CH, Jeong JK, Kim ES (2006). TTF-1, a homeodomain-containing transcription factor, regulates feeding behavior in the rat hypothalamus.. Biochem Biophys Res Commun.

